# Reactive lymphoid hyperplasia of the liver requires differential diagnosis of hepatocellular carcinoma

**DOI:** 10.1186/s40792-015-0034-4

**Published:** 2015-03-24

**Authors:** Takaaki Higashi, Daisuke Hashimoto, Hiromitsu Hayashi, Hidetoshi Nitta, Akira Chikamoto, Toru Beppu, Hideo Baba

**Affiliations:** Departments of Gastroenterological Surgery, Graduate School of Medical Sciences, Kumamoto University, 1-1-1 Honjo, Kumamoto-shi, Kumamoto 860-8556 Japan

**Keywords:** Reactive lymphoid hyperplasia, Primary biliary cirrhosis, Middle-aged women

## Abstract

Reactive lymphoid hyperplasia (RLH) of the liver is a rare and benign nodular lesion. It remains difficult to distinguish RLH from hepatocellular carcinoma (HCC) despite recent advances in imaging modalities. We report five cases of RLH that required differential diagnosis of HCC preoperatively. These cases all occurred in middle-aged women and were associated with autoimmune disease in 40% (2/5). The diameter of the nodule was less than 2 cm in all five of our cases. Four cases had a preoperative diagnosis of HCC. When a liver nodule is found in middle-aged women with an autoimmune disease, the possibility of RLH should be considered.

## Background

Reactive lymphoid hyperplasia (RLH) of the liver is a rare and benign nodular lesion that is characterized on histopathology by the proliferation of non-neoplastic lymphocytes forming follicles [1]. To the best of our knowledge, only 47 cases of hepatic RLH have been reported in the English literature. Herein, we report five cases of RLH of the liver and clarify the clinical features of this lesion from a review of other cases.

## Case presentation

### Patients’ characteristics

Five patients underwent hepatic resection for hepatocellular carcinoma (HCC) at the Department of Gastroenterological Surgery and Transplantation Pediatric Surgery Kumamoto University Hospital. All patients were female with a median age of 55 years (range 44 to 68). Written informed consent was obtained from all the patients before treatment. Median tumor size is 1.55 cm (range 0.96 to 1.8). Two of the five patients (40%) had a history of autoimmune disease which is primary biliary cirrhosis (PBC). All cases were suggested malignant disease, and hepatic resection was performed (Table 1).

**Table 1:** **Characteristics of our five cases**

**Case**	**Age**	**Sex**	**Child-Pugh (point)**	**Size (cm)**	**Preoperative diagnosis**	**Background of the liver**	**Therapy**
1	60	F	A (5)	0.96	HCC	Normal	Laparoscopic partial resection
2	68	F	A (5)	1.4	MALT lymphoma	Normal	Segmentectomy
3	52	F	B (8)	1.6	HCC	PBC	Transplantation
4	44	F	A (5)	2	HCC	Normal	Segmentectomy
5	51	F	A (5)	1.8	HCC	PBC	Partial resection

HCC, hepatocellular carcinoma; PBC, primary biliary cirrhosis.

### Preoperative findings

**Figure 1:**
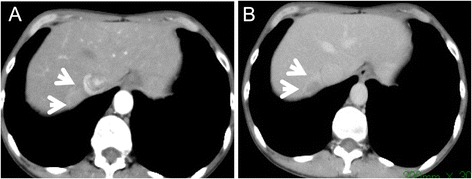
**CT demonstrated hypodense nodule.** Immediately enhanced in the early phase **(A)** and quickly de-enhanced in the late phase **(B)** after injection of contrast.

Computed tomography (CT) scan demonstrated hypodense nodules that were immediately enhanced in the early phase and quickly de-enhanced in the late phase after injection of contrast (Table 2 and Figure 1).

**Table 2:** **Preoperative findings in five cases**

**Case**	**CTA**	**CTAP**	**MRI T1-weighted images**	**MRI T2-weighted images**	**MRI arterial phase**	**MRI venous phase**	**ADC**
1	Enhance	De-enhance	Hypo	Hyper	Enhance	De-enhance	1.4
2	Enhance	De-enhance	Hypo	Hyper	ND	ND	1
3	Enhance	De-enhance	Hypo	Hyper	ND	ND	ND
4	Enhance	De-enhance	Hypo	Hyper	Enhance	De-enhance	1.6
5	Enhance	De-enhance	Hypo	Hyper	Enhance	De-enhance	1.1

CTA, computed tomography angiography; CTAP, computed tomography during arterial portgraphy; MRI, magnetic resonance imaging; ADC, apparent diffusion coefficient; ND, not determine.

**Figure 2:**
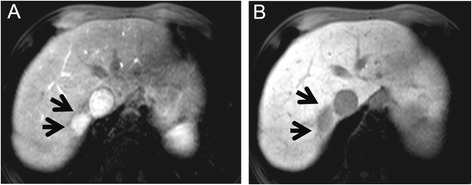
**Contrast MRI showed a hyperintense nodule and hypointense nodule.** A hyperintense nodule in the arterial phase **(A)** and a hypointense nodule in the hepato-biliary phase **(B)**.

Magnetic resonance imaging (MRI) showed a lower intensity nodule on T1-weighted images and a higher intensity nodule on T2-weighted images. Following injection of contrast, MRI produced images that were similar to those obtained with CT (Table 2 and Figure 2).

We calculated the apparent diffusion coefficient (ADC) in four patients, but the mean ADC was 1.25 (range 1 to 1.6), and this was similar to the ADC reported for HCC.

Since both the CT and MRI images suggested hepatocellular carcinoma or mucosa-associated lymphoid tissue (MALT) lymphoma, distant metastasis was not observed in all cases, and therefore, they underwent planned hepatic resection with their informed consent.

### Histological findings

**Figure 3:**
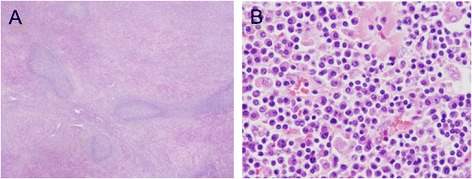
**Histopathology revealed a relatively well-circumscribed nodular proliferation of mature-appearing small lymphocytes with lymphoid follicles.** The lymphoid follicles varied in size **(A)**. The germinal centers were comprised of a mixture of small and large lymphoid cells with no nuclear atypia. Strands of amorphous, hyalinized material were observed in the interfollicular areas **(B)**.

In all the cases, tumors were diagnosed as RLH based on hematoxylin and eosin (HE) staining and immunohistochemistry (Figure 3). HE staining showed a relatively well-circumscribed nodular proliferation of mature-appearing small lymphocytes with lymphoid follicles. The germinal centers were comprised of a mixture of small and large lymphoid cells. Strands of amorphous, hyalinized material were observed in the interfollicular areas.

**Figure 4:**
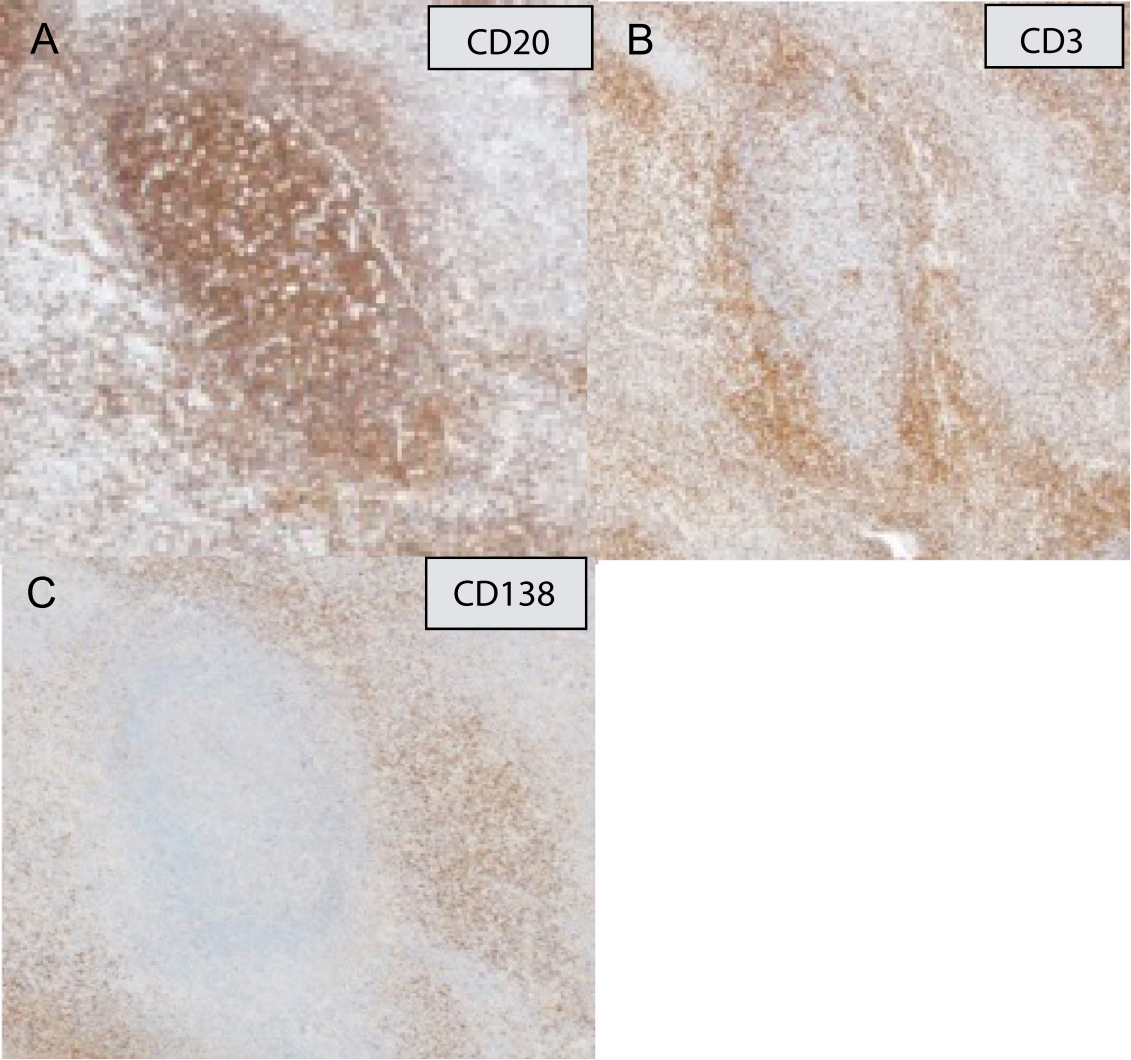
**Immunohistochemical studies.** The follicles were mainly comprised of CD20-positive lymphocytes **(A)**, while CD3-positive cells were distributed between the follicles and around the circumference of the follicles **(B)**. The stromal areas contained CD138-positive plasma cells **(C)**.

Immunohistochemical studies (Figure 4) showed that the follicles were mainly comprised of CD20-positive lymphocytes, whereas CD3-positive cells were observed between the follicles and around the circumference of the follicles. CD21-positive dendritic cells were distributed within the germinal center. The intrafollicular areas contained CD138-positive plasma cells.

## Discussion

Reactive lymphoid hyperplasia of the liver is an extremely rare condition, and only 47 cases of hepatic RLH have been reported in the English literature. In addition, we experienced five cases from 2003 to 2011 (Table 1). RLH is a benign nodular lesion mainly detected in the lung, stomach, small intestine, orbit, pancreas, skin, and breasts [2]. RLH occurs predominantly in middle-aged women with a mean age of 55 years [3]. The male-female ratio of hepatic RLH reported in the literature was 1:9.7 [3]. The diameter of the lesions ranged from 0.4 to 5.5 cm; however, most were less than 2 cm [4]. In our five cases, RLH occurred in women, and the diameter of the lesion was less than 2 cm. Although the pathogenesis remains unknown, an association between the development of hepatic RLH and systemic or local immunological abnormalities has been reported. Chronic liver diseases including HBV- or HCV-related liver cirrhosis were found in 27% of patients, and autoimmune disorders such as PBC were diagnosed in 23% [5]. In our five cases, 40% (2/5) had an autoimmune disorder (PBC). Chronic stimulation by autoimmune antigens may play a role in RLH associated with autoimmune disease [6].

**Figure 5:**
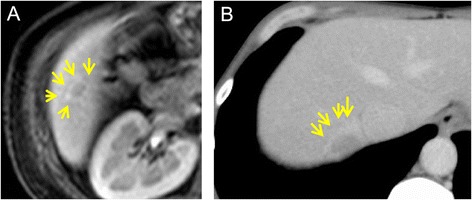
**Contrast MRI (A) and CT (B) showed perinodular enhancement in the equilibrium phase.**

Since RLH is a benign nodular lesion, preoperative diagnosis of hepatic RLH by clinical imaging would be useful to reduce unnecessary invasive procedures. However, preoperative differential diagnosis is extremely difficult because hepatic RLH has features similar to HCC on various imaging modalities [1,7,8]. Several imaging modalities in our cases showed that RLH could not be distinguished from HCC (Table 1). Diffusion-weighted MRI is sensitive to molecular diffusion and allows for tissue characterization by probing tissue microstructural changes. While the ADC [9] has been demonstrated to be useful to distinguish malignant from benign lesions [10], the mean ADC in our patients was 1.25 (range 1 to 1.6) and this was similar to the ADC reported for HCC. However, making a diagnosis of RLH by using imaging studies alone is challenging, there is an interesting report to describe a unique finding in RLH [11]. It was shown that the multiphasic contrast-enhanced CT and MRI findings included a fact, which is ‘perinodular enhancement,’ on arterial or equilibrium phase. In fact, to focus on the perinodular area in our cases, perinodular enhancement was observed on equilibrium phase (Figure 5). If we found a single and small (≦2 cm) tumor in female patients who has no risk factors of HCC, the possibility of RLH should be considered. Additionally, the careful observation of contrast-enhanced CT and MRI, particularly the area of perinodular area, may lead to a diagnosis of RLH.

## Conclusions

We report five cases of hepatic RLH requiring differential diagnosis from HCC and MALT lymphoma. It is extremely difficult to diagnose RLH preoperatively because it has features similar to HCC on various imaging modalities. When a liver nodule is found in middle-aged women with an autoimmune disease, the possibility of RLH should be considered.

## Consent

Written informed consent was obtained from the patient for publication of this case report and any accompanying images. A copy of the written consent is available for review by the Editor in Chief of this journal.

## Abbreviations

ADC: apparent diffusion coefficient
CT: computed tomography
CTA: computed tomography angiography
CTAP: computed tomography during arterial portgraphy
HCC: hepatocellular carcinoma
HE: hematoxylin and eosin
MRI: magnetic resonance imaging
ND: not determine
PBC: primary biliary cirrhosis
RLH: reactive lymphoid hyperplasia
